# An Improved Pansharpening Method for Misaligned Panchromatic and Multispectral Data

**DOI:** 10.3390/s18020557

**Published:** 2018-02-11

**Authors:** Hui Li, Linhai Jing, Yunwei Tang, Haifeng Ding

**Affiliations:** Key Laborary of Digital Earth Sciences, Institute of Remote Sensing and Digital Earth, Chinese Academy of Sciences, Beijing 100094, China; tangyw@radi.ac.cn (Y.T.); dinghf@radi.ac.cn (H.D.)

**Keywords:** pansharpening, high-resolution remote sensing, spectral distortion, misalignment

## Abstract

Numerous pansharpening methods were proposed in recent decades for fusing low-spatial-resolution multispectral (MS) images with high-spatial-resolution (HSR) panchromatic (PAN) bands to produce fused HSR MS images, which are widely used in various remote sensing tasks. The effect of misregistration between MS and PAN bands on quality of fused products has gained much attention in recent years. An improved method for misaligned MS and PAN imagery is proposed, through two improvements made on a previously published method named RMI (reduce misalignment impact). The performance of the proposed method was assessed by comparing with some outstanding fusion methods, such as adaptive Gram-Schmidt and generalized Laplacian pyramid. Experimental results show that the improved version can reduce spectral distortions of fused dark pixels and sharpen boundaries between different image objects, as well as obtain similar quality indexes with the original RMI method. In addition, the proposed method was evaluated with respect to its sensitivity to misalignments between MS and PAN bands. It is certified that the proposed method is more robust to misalignments between MS and PAN bands than the other methods.

## 1. Introduction

Due to the physical constraints of remote sensing imaging and limited bandwidth of satellite transmission, a large number of currently operating satellites, such as SPOT, IKONOS (IK), QuickBird (QB), and WorldView-2/3, provide both a single relative high-spatial-resolution (HSR) panchromatic (PAN) band and several low-spatial-resolution (LSR) MS bands. Fusion of PAN and MS images is also referred to as pansharpening [1]. It is an important pre-processing step for generating high quality HSR MS image in various remote sensing tasks [2,3], such as land cover classification, object recognition [4,5], water-bodies mapping [6,7,8], and shadow detection [9]. In recent years, the requirement for fused HSR MS imagery is unceasingly growing, due to abundant remote sensing data sources [10].

Research on pansharpening has been done for decades and numerous algorithms have been developed to produce HSR MS imagery. The major problem encountered by current pansharpening methods is to reduce spectral and spatial distortions. Researchers have proposed several categorizations to group existing pansharpening methods [11,12,13,14,15,16]. One of the categorizations classifies current pansharpening methods into two categories: the component-substitution (CS) methods and the methods based on multiresolution analysis (MRA) [17,18,19,20]. The CS methods can provide fused products with good spatial quality in most cases, but they sometimes suffer from spectral distortions. The MRA-based methods can provide fused products with good spectral quality. However, their fused products may suffer spatial distortions [21]. Moreover, this disadvantage may be emphasized for misaligned MS and PAN data, especially for MSA-based methods using transformations that are not shift-invariant to achieve multiresolution analysis [21,22]. In contrast, the performances of CS methods are robust to possible misalignment between MS and PAN bands. In addition, they have a relative low computational burden. These favorable characteristics lead to their widespread uses.

During the designing of pansharpening algorithms, it is commonly assumed that PAN and MS bands are perfectly aligned. Researchers have made tremendous progress in image registration, for both nature images [23,24,25] and remote sensed images [26,27]. However, it is hard to reach perfect co-registration between PAN and MS bands. Even through PAN and MS images are often simultaneously acquired by the same platforms, there may be misalignment, due to small angle difference between the two sensors exists in data acquisition process. Hence, some misregistration between PAN and MS images is unavoidable in the real world. The impact of image misalignment on quality of fused products has been reported by several researchers. Blanc et al. [28] showed that even a small misregistration of 0.3 pixel can cause a noticed effect on quality of fused images. Baronti et al. [29] theoretically analyzed the effects of misregistration on pansharpened images and concluded that misregistration degrades the performances of all pansharpening methods. In addition, MRA-based methods are much more sensitive to registration errors than CS methods. In order to evaluate quality of pansharpened images with reference to a true MS image, original MS and PAN datasets are usually spatially degraded to a lower resolution. Jing et al. [30] reported that misalignments between MS and PAN bands can be caused by some decimation approaches used to produce degraded datasets, and thus lead to undependable performance evaluation of different pansharpening methods. Xu et al. [13] discussed two key problems that affect quality of fused images, i.e., misregistration and size difference caused by different resolutions between MS and PAN bands. It was found that the two problems should be considered during the design of a fusion algorithm. Hallabia et al. [31] proposed a modified pansharpening method based on filter banks, taking into account the case of misalignment. Jing and Cheng [18] proposed a ratio-based method that can reduce misalignment impact, which is denoted as RMI. It was demonstrated that the RMI method can provide good performances, in terms of quality indexes, as well as visual inspection, for both aligned and misaligned PAN and MS images [18]. This method can achieve good performances, due to two reasons. The key reason is that it uses a synthetic PAN band at PAN scale generated with weighted sum of up-sampled MS bands using optimal regression coefficients obtained at MS scale. The second one is that this method also considers to reduce the effect of image haze caused by atmospheric path radiance.

In this paper, an improved version of the RMI method is proposed. Two improvements were made. The first one is that pixels corresponding to edge pixels identified from the PAN bands are fused through the injection of spatial details with relative large coefficients, to sharpen boundaries between different image objects. The second one is that dark pixels are fused using haze values lower than those used to other pixels, in order to improve the quality of fused dark pixels. The experimental results showed that the proposed method can reduce spectral distortions of fused dark pixels, and sharpen boundaries between different objects. Meanwhile, it is robust to misalignments between MS and PAN bands. The rest of this paper is organized as follows: the proposed improved method is introduced in Section 2, a fusion experiment is reported in Section 3. Section 4 presents some related discussions, whereas Section 5 summarizes the conclusions of this work.

## 2. Methodologies

### 2.1. The RMI Method

The RMI method is a ratio-based approach considering haze caused by atmospheric path radiances. Let *H_P_* and *H_i_* denote image haze values determined for PAN and *i*th MS bands, respectively, *I*_i_ denotes the *i*th band of the up-sampled MS image, and *P* and *P_S_* are an original PAN band and a synthetic version of the original PAN band, respectively. The fused *i*th MS band *F*_i_ generated by the RMI method can be expressed using Equation (1):

(1) $$F_{i}=\left(I_{i}-H_{i}\right)\frac{P-H_{p}}{P_{S}-H_{p}}+H_{i},\;i=1,\cdots ,\;N$$

Equation (1) can also be rewritten as (2):

(2) $$F_{i}=I_{i}+\frac{I_{i}-H_{i}}{P_{S}-H_{p}}\left(P-P_{S}\right),\;i=1,\cdots ,\;N$$

in which *P* − *P*_S_ represents spatial details derived from the original PAN band, and $\frac{I_{i}-H_{i}}{P_{S}-H_{p}}$ is the space-varying injection coefficients for the *i*th band.

*P_S_* is obtained by a weighted sum the up-sampled MS bands, as in (3):

(3) $$P_{S}=\overset{N}{\underset{i=1}{\sum }}a_{i}\cdot I_{i}+b$$

in which *a_i_* is the weight coefficient of the *i*th band, and *b* is a constant. The optimal values of *a_i_* and *b* can be estimated by the least-squares approach using Equation (4):

(4) $$P_{L}=\overset{N}{\underset{i=1}{\sum }}a_{i}\cdot I_{i}^{L}+b$$

where *P_L_* is a degraded version of the original PAN band, and can be obtained using an averaging approach; $I_{i}^{L}$ is the *i*th original MS band.

For misaligned MS and PAN bands, the optimal regression coefficients, representing the maximum multiple correlations between misaligned PAN with each MS band, can discount the effect of misalignment on quality of fused images. Another factor contributing to the good performance of the RMI method is that image haze caused by atmospheric path radiance is considered by the method. However, haze values of PAN and MS bands give an important effect on spectral vector directions of fused MS pixels and thus the spectral quality of the whole fusion product [32].

### 2.2. The Improved Version of the RMI Method

In this work, two improvements are made based on the original RMI method. For the first improvement, boundary pixels of the PAN band are identified using edge detection operators, and then fused using larger injection coefficients than the other pixels. Fused images generated by commonly used methods show blurred boundaries between different objects, due to several reasons, such as misalignments, aliasing, and the effect of mixed pixels. As this improvement aims to add more spatial details into edge pixels, it helps to obtain fused products with sharpened boundaries between different image objects. For an edge pixel *t*, the fusion version of *t* is calculated using (5):

(5) $$F_{i}\left(t\right)=I_{i}\left(t\right)+\left(1+k/10\right)\cdot \frac{I_{i}\left(t\right)-H_{i}}{P_{S}\left(t\right)-H_{p}}\left[P\left(t\right)-P_{S}\left(t\right)\right],\;i=1,\cdots ,\;N,k\;=\;0,\;1,\cdots ,4$$

in which *k* is a value determining the sharpness boundaries between different objects in pansharpened products. The value can be set by users, according to different application purposes.

For the second improvement, relative dark pixels in the image, which mainly correspond to dark objects such as water-bodies and shadows, are identified according to a certain threshold and then fused using relative low haze values. As a high haze value may result in abnormal high injection coefficients for dark pixels, this may result in over-enhancement of dark pixels. This improvement is helpful for reducing spectral distortions of water-bodies and shadow covered regions in fused products. In this work, a threshold *T*, used to justify dark pixels, were automatically determined using the product of the standard deviation of the PAN band *δ_P_* and a scale factor *S*.

For a pixel with a gray value higher than *T* in PAN, the fused version $F_{i}$ can be produced using (2). In contrast, for a pixel with a gray value lower than *T* in PAN, relative low haze values for MS and PAN bands, which are denoted as $H_{i}^{L}$ and $H_{P}^{L}$, respectively, are employed to generate fused pixels. In this situation, the fused version $F_{i}$ can be produced using (6):

(6) $$F_{i}=I_{i}+\frac{I_{i}-H_{i}^{L}}{P_{S}-H_{P}^{L}}\left(P-P_{S}\right),\;i=1,\cdots ,N$$

in which $H_{i}^{L}$ is determined using (7):

(7) $$H_{i}^{L}=p\cdot H_{i},\;i=1,\cdots ,\;N$$

where *p* is a scale constant be lower 1. A value of 0.75 was assigned to *p* in this work. The values of $H_{P}^{L}$ are obtained with respect to coefficients obtained using (4), as in (8):

(8) $$H_{P}^{L}=\sum_{i=1}^{N}a_{i}\cdot H_{i}^{L}+b.$$

The proposed method is implemented in MATLAB. The pseudocode of the proposed method is also reported, as Algorithm 1, to facility other people to implement and use the method in remote sensing tasks.

 **Algorithm 1:** Improved_RMI   **input:** upsampled MS bands *I*, PAN band *P*, band number *N*   **output:** fused MS bands *F*    Let *P_L_* be a PAN band at MS scale, generating from *P* using an averaging approach   Let *a_i_* and *b* are the coefficients generated using (4) by the least-squares approach   PS ← $\sum_{i=1}^{N}a_{i}\cdot I_{i}+b$   Let *H_p_* and *H_i_* be haze values for PAN and the *i*th MS bands, respectively   Let $H_{i}^{L}$ and $H_{P}^{L}$ be haze values for dark pixels   $H_{i}^{L}$ ← $p\cdot H_{i}$   $H_{P}^{L}$ ← $\sum_{i=1}^{N}a_{i}\cdot H_{i}^{L}+b$   Let *E* be the union of the edge PAN pixels identified using CANNY, let *F* be the other pixels no belonging to *E*   Let *F_i_* be the fused *i*th MS band   **for each** pixel *t*
**in**
*E*      **for each** band *i* in [1, 2, …, *N*]           *F_i_(t)* is calculated using (5)      **end for**   **end for**    **for each** pixel *t*
**in**
*F*      Let *T* be the threshold used to identify dark pixels, determined using *T* = *δ_P_* × *S*      **for each** band *i* in [1, 2, …, *N*]           **if**
*P(t)* − *H_p_* ≥ *T*                *F_i_(t)* is calculated using (2)           **Else**                *F_i_(t)* is calculated using (6)           **end if**      **end for**   **end for**   **return**
*F*

## 3. Experiments

### 3.1. Test Data

**Figure 1:**
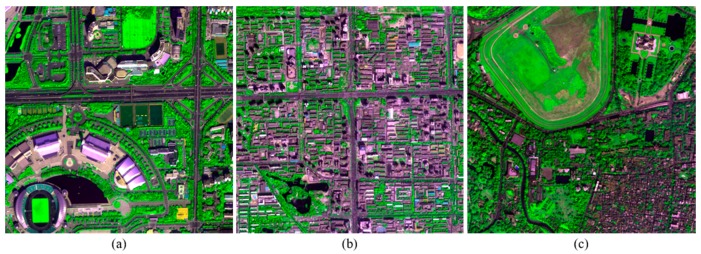
The MS images of the WV2 (**a**); IK (**b**); and QB (**c**) datasets.

Three image scenes recorded by WorldView-2 (WV2), IK, and QB, respectively, were used in the experiment to evaluate the performances of the proposed method. A subset with a size of 512 × 512 pixels for MS bands, and a size of 2048 × 2048 pixels for PAN bands, were selected from each scene. The MS image from WV2 has eight bands, whereas those of IK and QB have four bands. The spatial resolution ratio *R* for all the three datasets is 4. The MS images of three datasets are presented in Figure 1.

### 3.2. Comparing with Other Methods

#### 3.2.1. Fusion Methods for Comparison and Evaluation Criteria

The proposed method was compared with adaptive Gram-Schmidt (GSA) [5,33], and Generalized Laplacian Pyramid (GLP) with Gaussian-shaped filter adjusted by modulation transfer function using spectral distortion minimizing model [34]. Actually, image haze can also be considered by some other methods using similar injection models. In order to achieve impartial comparison, the GLP method considering image haze was also achieved and included in the comparisons. It is noted as GLP-H henceforth. The GSA method with image haze was also considered, but it was not included in this work because it yielded the same performance as the original GSA method. Actually, it can be inferred that the considering of image haze could not improve or decrease the performance of GSA.

Besides the original versions of the three datasets, the degraded versions of the original datasets were also considered, as fused images generated at the degraded scale can be assessed using the original MS images as reference. Quality of fused products of the degraded scale was assessed using several widely used quality indexes including the relative average spectral error (RASE) [35], dimensionless global relative error of synthesis (ERGAS) [36], spectral angle mapper (SAM) [37], Q2^n^ [38,39,40], and spatial correlation coefficient (SCC) [41]. An index named quality with no reference index (QNR) [42], which is increasingly used in recent studies, was employed to evaluate quality of pansharpened images produced using the original datasets. The QNR index is a comprehensive index consisting of a spectral distortion indicator D_λ_ and a spatial distortion indicator D_S_ [42]. In addition, visual inspection was also performed to comparing the quality of fused products. In order to avoid misalignments between MS and PAN bands, an aligned version of an original PAN band was determined using the similar approach employed in [43], and then used to replace the original PAN bands in this experiment. The degraded MS and PAN images were generated by a box filter with a size of 4 × 4, according to the resolution ratio of the three datasets, in order to avoid misalignment introduced during the decimation process [30].

#### 3.2.2. Results and Analysis 

Table 1 reports the quality indexes generate from the fused products obtained at the two scales. The improved methods with different values for *k* used the same haze values as those employed by the original RMI method, for each test image. In this table, RMI (*k* = *n*) denotes the fused images generated by the RMI method with *k* = *n*, where *n* is an integer ranging from 0 to 4. In order to highlight the performances of the comparing methods on the fusion of dark pixels, a SAM value between fused and reference dark pixels is calculated from each fused image generated at the degraded scale. Version and the original version of dark pixels, which are identified during the implement of the proposed method. This index is also listed in Table 1, and it is denoted as SAM_d_ in the table.

It can be observed from Table 1 that RMI (*k* = 0) gives slightly better or similar performances with the original RMI method. The improved and original RMI methods provide higher Q2^n^ and QNR values than other methods. An exception is that GSA and GLP-H yield lower D_λ_ and D_s_ values, and higher QNR values than other methods for the original QB dataset. For the degraded WV2 dataset, RMI (*k* = 0) offers slightly lower RASE and ERGAS values, and slightly higher Q2^n^ and SCC values than the original RMI method. For the original WV2 dataset, RMI (*k* = 0) provides a slightly lower D_s_ value and a slightly higher QNR value than the original RMI method. For the degraded IK dataset, RMI (*k* = 0) provides slightly lower Q2^n^ and RASE values than the original RMI method, and the same ERGAS and SAM values as the original RMI. The former offers a slightly higher QNR value and a slightly lower D_s_ value than the latter, for the original IK dataset. For the degraded QB dataset, RMI (*k* = 0) provides slightly lower RASE, ERGAS, SAM values, and higher Q2n and SCC values than the original RMI. For the original QB dataset, RMI (*k* = 0) also yields a lower D_λ_ and a higher QNR than the original RMI. The improved RMI methods offer lower SAM_d_ than the original RMI for all the three datasets, indicating that the proposed method is effective for reducing spectral distortions of fused dark pixels. The GSA method yields the highest SAM_d_ values for the three degraded datasets, indicating it gives the poorest performances for dark pixels. As the three test images have different numbers of dark pixels, it is reasonable that the proposed approach gives slightly different performances for these images.

**Figure 2:**
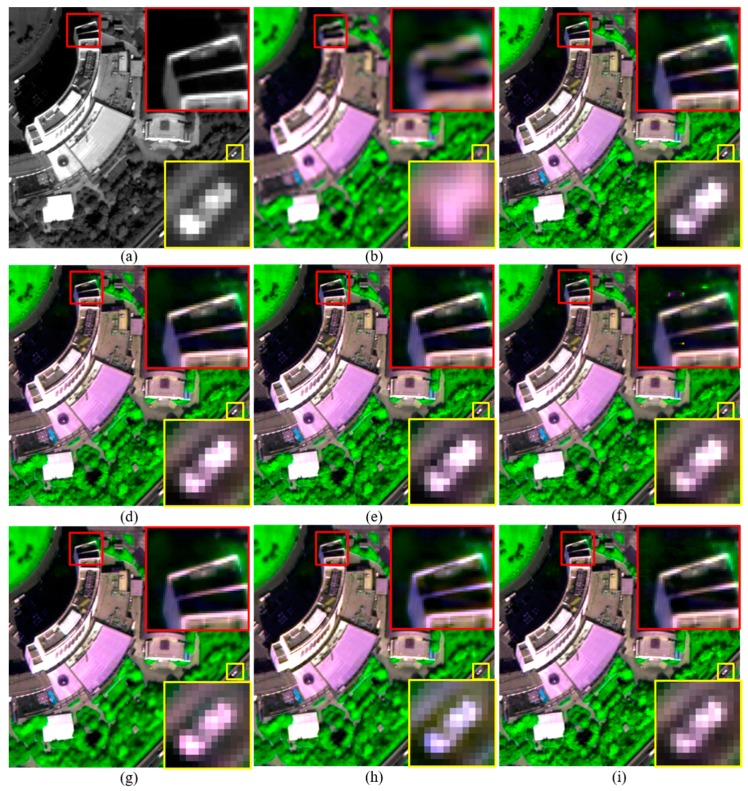
The original and pansharpened images of a 400 × 400 subset from the original WV2 dataset. (**a**) 0.5-m PAN; and (**b**) the up-sampled version of 2-m MS; and fused images generated by the (**c**) RMI (*k* = 0); (**d**) RMI (*k* = 2); (**e**) RMI (*k* = 4); (**f**) RMI; (**g**) GSA; (**h**) GLP; and (**i**) GLP-H methods.

**Figure 3:**
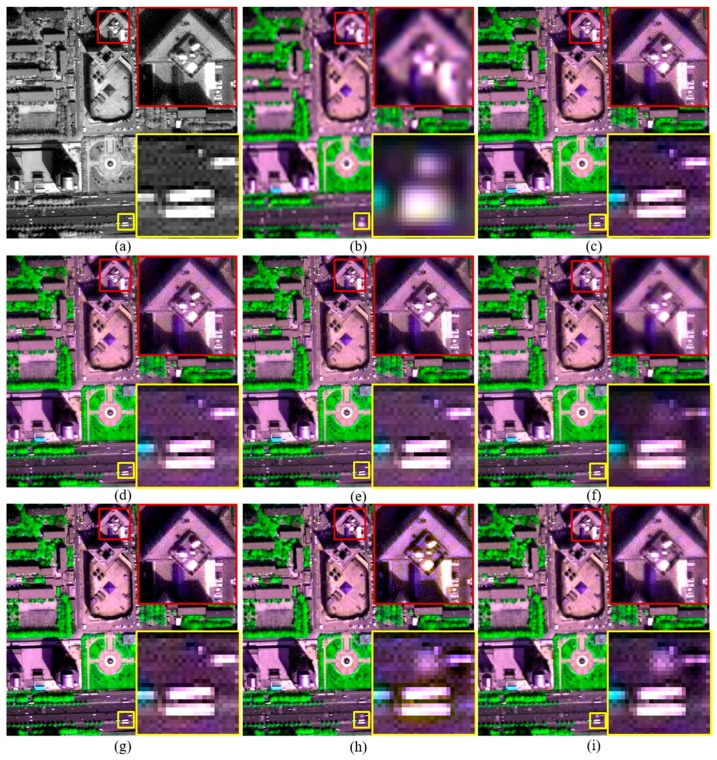
The original and pansharpened images of a 400 × 400 subset from the original IK dataset. (**a**) 1-m PAN; and (**b**) the up-sampled version of 4-m MS; and fused images generated by the (**c**) RMI (*k* = 0); (**d**) RMI (*k* = 2); (**e**) RMI (*k* = 4); (**f**) RMI; (**g**) GSA; (**h**) GLP; and (**i**) GLP-H methods.

**Figure 4:**
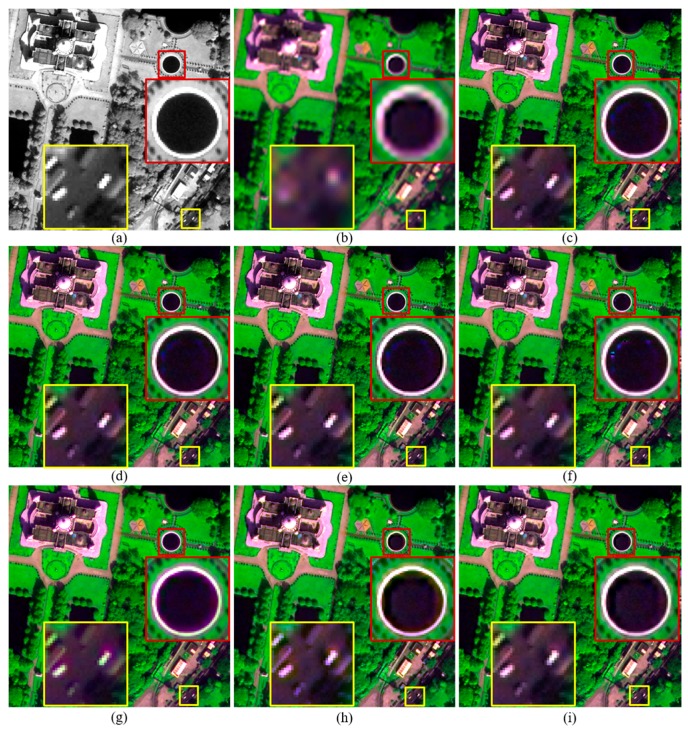
The original and pansharpened images of a 480 × 480 subset from the original QB dataset. (**a**) 0.7-m PAN; and (**b**) the up-sampled version of 2.8-m MS; and fused images generated by the (**c**) RMI (*k* = 0); (**d**) RMI (*k* = 2); (**e**) RMI (*k* = 4); (**f**) RMI; (**g**) GSA; (**h**) GLP; and (**i**) GLP-H methods.

The Q2^n^ and QNR values of fused products generated by the improved RMI methods descend along with the increasing of *k*. However, the fused products generated by RMI (*k* = 4) also offer higher Q2^n^ and QNR values than those generated by the GSA, GLP, and GLP-H methods for the WV-2 and IK datasets. The GLP-H method offers higher Q2^n^ and QNR values than GSA and GLP for the three datasets, due to the considering of image haze. The GLP method offers lower Q2^n^ and QNR values than GSA for the three datasets. This may due to the fact that misalignments between MS and PAN bands occur during an down-sampling progress for obtaining a LSR PAN band at MS scale, and an followed up-sampling progress for obtaining an expanded version of the LSR PAN band.The original and pansharpened images of a subset selected from each original dataset are shown in Figure 2, Figure 3 and Figure 4, for visual inspection. For the WV-2 dataset, the images are shown in compositions of bands 5, 7, and 2. For the IK and QB datasets, the images are shown in compositions of bands 3, 4, and 1.

Although pansharpened images generated by these methods show similar tone in each figure, fused images produced by the improved and original RMI methods, and GLP-H show more texture details in vegetation covered regions. This is obvious in Figure 2. This is due to the considering of image hazes by these methods. Some artefacts can be seen from the fused images generated by the original RMI method, as shown in Figure 2f and Figure 4f. The artefacts occur in some regions covered by shadows or water-bodies. In contrast, these artefacts are absent from the pansharpened images generated by RMI (*k* = 0), RMI (*k* = 2), and RMI (*k* = 4), as shown in Figure 2 and Figure 4. Although GSA yields the highest QNR value for the original QB dataset, the GSA-fused images show obvious spectral distortions. This can be observed from the yellow rectangle in Figure 2g, and the red and yellow rectangles in Figure 4g. In addition, the GSA-fused WV2 image show very few texture details for vegetation covered regions. This is because a very small amount of spatial details is injected into the two near-infrared (NIR) bands of the WV2 dataset, due to relative low correlations between PAN and the two NIR bands. The fused images generated by GLP and GLP-H also show noticeable spectral distortions, as shown in yellow rectangles in Figure 2, Figure 3 and Figure 4. Moreover, aliasing effects can be observed in the fused images generated by GLP and GLP-H, as shown in yellow rectangles in Figure 3 and Figure 4. Moreover, the fused products produced by RMI (*k* = 2), and RMI (*k* = 4) offer more sharpened boundaries between different objects than the original RMI, GSA, GLP, and GLP-H. This can be seen from the fused subsets in yellow rectangles shown in Figure 2, Figure 3 and Figure 4. The fused images generated by RMI (*k* = 2), and RMI (*k* = 4) provide more sharpened boundaries than those produced by RMI (*k* = 0), due to more spatial details are injected into edge pixels. Consequently, the improved approach can reduce spectral distortions of fused dark pixels and sharpen some boundaries in fused products, as well as obtain similar quality indexes with the original RMI method.

### 3.3. Sensitivity to Misalignments between MS and PAN Bands

The proposed method is also evaluated with respect to its sensitivity to misalignments between MS and PAN bands using another experiment, with the degraded IK dataset. The up-sampled version of the degraded MS image was shifted by zero to four PAN pixels on row and column, respectively, to yield several shifted versions of the up-sampled MS image. A shift of *r* pixels on row, along with *c* pixels on column, is denoted as a couple (*r*, *c*). Several couples including (0, 1), (1, 1), (2, 1), (2, 2), (3, 2), (3, 3), (4, 3), and (4, 4) were employed to generated shifted MS images. These shifted up-sampled MS images were then fused with the degraded 4-m PAN band using the proposed method, GSA, GLP, and GLP-H, to produce 4-m fused products.

**Figure 5:**
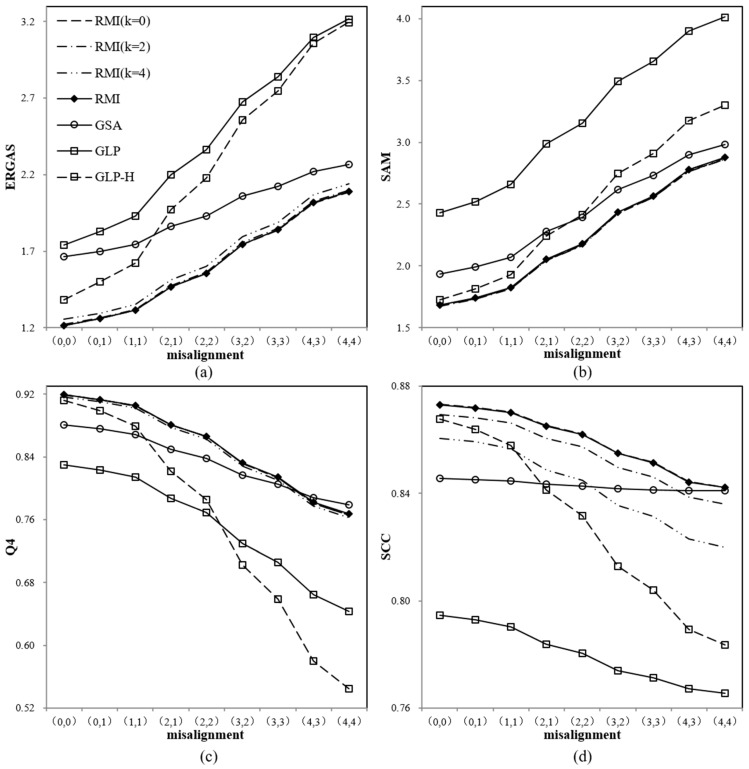
Variations of the ERGAS (**a**); Q4 (**b**); SAM (**c**); and SCC (**d**) indices of pansharpened images produced from the degraded IK dataset with different misalignments between PAN and MS bands.

The quality of these fused products was also assessed using ERGAS, SAM, Q4, and SCC, which are shown in Figure 5a–d, respectively. As the improved RMI methods with different *k* values give very clustered indexes, only the cases including *k* = 0, *k* = 2, *k* = 4 are shown in the figure for better visual inspection. As illustrated in those figures, the original RMI, RMI (*k* = 0), and RMI (*k* = 2) offer very similar performances, in terms of the four indexes. Actually, RMI (*k* = 0) offers slightly lower ERGAS and SAM values, and slightly higher Q4 and SCC values, than the original RMI method. RMI (*k* = 2) also gives lower SAM values than the original RMI method.

The pansharpened images generated by the original RMI, RMI (*k* = 0), and RMI (*k* = 2) offer the lowest ERGAS and SAM values, and the highest Q4 and SCC values, in most cases. Exceptions occur when the misalignments are (4, 3) and (4, 4), the GSA method yields the highest Q4 values, and higher SCC values than the RMI (*k* = 2). In addition, RMI (*k* = 4) offers lower SCC values than the GSA and GLP-H methods in some cases, although the former offers lower ERGAS and SAM values for all the cases. This indicates that RMI (*k* = 0), and RMI (*k* = 2) are the best choices, when misalignments between MS and PAN bands are no more than three PAN pixels on row and column.

The GSA method gives better performances than the two GLP methods when the misalignment is larger than two pixels. However, when the misalignment is no more than one pixel, the GSA method offers higher ERGAS values and lower Q4 values than the GLP-H method.

The GLP-H method yields lower ERGAS and SAM values, and higher SCC values than the GLP method for all the cases. However, it offers higher Q4 values than the GLP and GSA methods when the misalignment is not more than two pixels, lower Q4 values than the latter two when the misalignment is larger. This indicates that the GLP-H gives better performances than GLP and GSA when the misalignment is not more than two pixels.

It can also be observed that the four quality indexes decline at different rates, as the misalignment increases. The four quality indexes of the original RMI, RMI (*k* = 0), RMI (*k* = 2), RMI (*k* = 4), and GSA decrease the more slowly than GLP and GLP-H. This is due to the fact that the MRA methods are more sensitive to misalignments between MS and PAN bands than the CS methods. Although the curves of GSA are even gentler than those of the original RMI, RMI (*k* = 0), RMI (*k* = 2), and RMI (*k* = 4), GSA offers poor performances than the latter in terms of quality indexes in most cases. In conclusion, the proposed method is also robust to misalignments between MS and PAN bands, besides offering comparable quality indexes to other methods.

**Figure 6:**
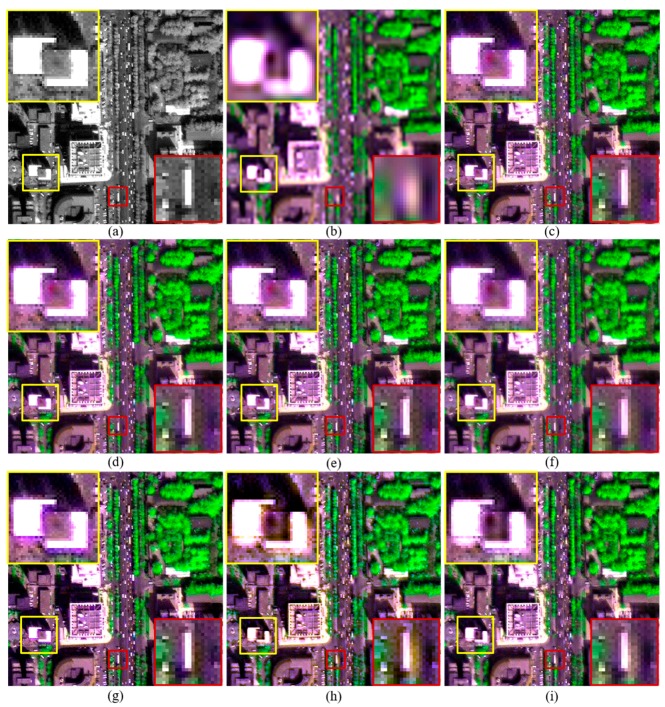
The original and fused images for a 300 × 300 subset of the original IK dataset with a misalignment of (2, 1). (**a**) 1-m PAN; and (**b**) the up-sampled version of 4-m MS; and fused images generated by the (**c**) RMI (*k* = 0); (**d**) RMI (*k* = 2); (**e**) RMI (*k* = 4); (**f**) RMI; (**g**) GSA; (**h**) GLP; and (**i**) GLP-H methods.

The original and fused images of a 300 × 300 subset of the original IK dataset with a shift of (2, 1) are shown in Figure 6. All the fused MS images were stretched to the same histogram as the corresponding original MS bands. It can be observed that the three fused products generated by RMI (*k* = 0), RMI (*k* = 2), RMI (*k* = 4), shown in Figure 6c–e, yield the best visual quality, followed by those produced by the original RMI and GSA. It can be observed from the yellow rectangle in Figure 6g that the GSA-fused image shows noticeable spectral distortion. In addition, aliasing effects can be observed from the fused images generated by GLP and GLP-H, as shown in the red and yellow rectangles in Figure 6h,i. Hence, visual inspection also indicates that the improved RMI is effective for reducing the impacts of misalignments between PAN and MS bands on quality of pansharpened products.

## 4. Discussion

The effects of misalignment on pansharpened RS images draw an increasing concern in recent years. Although the RMI method is been proposed for several years, the advantage of this method for fusion of misaligned MS and PAN bands is not explored yet. In addition, image haze is a non-negligible factor for ratio-based fusion methods, especially when image fusion is performed on remote sensing images in digital number. The determination of haze values gives an important impact on spectral distortion of fused products [32,43,44], especially for fused pixels corresponding vegetation and dark objects, such as shadows and water-bodies. This work proposed an improved version of the RMI method though two improvements. The quality indexes for the proposed method, which aims at reducing spectral distortions of fused dark pixels, did not show significant improvement in the experiment, due to a limited amount of dark pixels included by the test datasets. However, the proposed method shows consistent improvements in terms of SAM_d_, which is an SAM index calculated using only dark pixels. It was shown that the improved RMI method is also robust to misalignment between MS and PAN bands, as well as allowing to produce pansharpened images with sharped boundaries. This is very useful for producing high-resolution MS images covering urban regions. It is also helpful for local region mapping, image interpretation, and applications related to water-bodies and shadows. As the proposed method yield rich texture details in vegetation covered regions, it can provide comparable performances for remote sensing images covering agricultural areas and forest areas. The proposed method also can be used to medium resolution images, i.e., ASTER, Landsat 7/8, and Sentinel 2A. However, it shows more advantages for high-resolution images, as boundary information and texture details are richer in high-resolution images than in medium resolution images.

A major difference between the proposed method and other methods is that it allows producing fused products with more sharpened boundaries, through the determination of different values for *k*. In order to give some guidelines on how to choose the optimal value for this parameter, a fusion experiment was performed to test values ranging from 0 to 10 for *k*, using the degraded QB dataset. The quality indexes calculated from the fused images generated by the proposed method are reported in Table 2. It can be seen that the values of the Q2^n^ index descend with the increase of *k*. This is because more spatial details injected into the up-sampled MS bands may results in larger spectral distortion of fused products. As RMI (*k* = 4) provides comparable indexes to other methods (as shown in Table 1), such as GSA and GLP, we suggest that the maximum value for *k* can be set to 4, for the proposed method. We think this value can yield a better balance between spectral and spatial quality of fused pixels corresponding to edge PAN pixels.

As introduced in Section 2.2, a threshold used to justify dark pixels were automatically determined using the product of the standard deviation of the PAN band and a scale factor *S*. We used a value of 0.3 for *S* in the experiments in this work. In order to evaluate the impact of this parameter on performances of the proposed method, values ranging from 0.1 to 1 with a step of 0.1 were tested for *S* in another experiment. The degraded QB dataset was used in this experiment, and the value of *k* is set to 0. The quality indexes obtained from fused images generated by the proposed method are shown in Table 3. The proposed method with *S* = 0.2 yields the highest Q2^n^ value. It can be seen that the performance of the proposed method gets poor along with the increases of *S* from 0.2. However, the changes are not significant. The fused images generated using *S* values ranging from 0.1 to 0.4 offer very similar Q2^n^ values. We suggest that the value of *S* can be set to 0.2, or 0.3, according to the results of this experiment.

In this work, edge pixels of the PAN band were automatically identified using the CANNY detector with sensitivity thresholds that are automatically chosen. The proposed method may yield better performances if an optimal sensitivity threshold is selected for each dataset.

## 5. Conclusions

An improved pansharpening method considering image haze caused by atmospheric path radiance is proposed in this study, in order to further reduce spectral distortion of fused dark pixels and sharpen boundaries between different image objects. The improved method was compared with the original RMI, GSA, GLP, and GLP-H. The main conclusions derived from the experimental results are as follows:

(1) The improved approach can reduce spectral distortions of fused dark pixels, thus the proposed method is a good choice for producing high-resolution MS images in applications related to water-bodies and shadows.
(2) The improved approach can be used to obtain fused images with sharpened boundaries between different objects, through choosing a reasonable value for *k*. This is very useful for fused products covering urban regions, and fused products used for local region mapping or image interpretation.
(3) The experiment used to evaluate the sensitivities of these method to misalignments between MS and PAN bands showed that the proposed method is more robust to misalignments between the MS and PAN bands than the other methods. These conclusions indicate that the improved method is very promising to be widely used in practical remote sensing applications.

## Figures and Tables

**Table 1:** Quality indexes for fused products at the two scales. Numbers in bold indicate the best performances for each quality index along each dataset.

Image	Method	Degraded Scale						Original Scale		
		RASE	ERGAS	SAM	Q2^n^	SCC	SAM_d_	D_λ_	D_S_	QNR
WV2	RMI (*k* = 0)	**6.67**	**1.740**	**2.28**	**0.9360**	**0.845**	1.127	**0.0600**	**0.067**	**0.877**
	RMI (*k* = 1)	6.72	1.75	2.28	0.935	0.843	1.127	0.061	0.068	0.876
	RMI (*k* = 2)	6.80	1.77	2.29	0.934	0.840	1.127	0.062	0.068	0.875
	RMI (*k* = 3)	6.92	1.80	2.30	0.933	0.836	1.126	0.062	0.068	0.874
	RMI (*k* = 4)	7.05	1.84	2.31	0.931	0.830	1.126	0.063	0.068	0.873
	RMI	6.68	1.745	2.28	0.9357	0.844	1.148	**0.0600**	0.068	0.876
	GSA	7.60	1.98	2.69	0.913	0.824	2.204	0.074	0.089	0.844
	GLP	8.05	2.06	2.94	0.845	0.807	1.263	0.113	0.110	0.789
	GLP-H	7.48	1.95	2.36	0.932	0.843	**1.094**	0.066	0.066	0.872
	EXP	12.61	3.26	2.94	0.791	0.441	1.263	0.000	0.068	0.932
IK	RMI (*k* = 0)	**4.63**	**1.214**	**1.677**	0.9192	**0.8734**	0.577	**0.0556**	**0.0919**	**0.8576**
	RMI (*k* = 1)	4.65	1.22	1.67	0.919	0.872	0.577	0.057	0.093	0.855
	RMI (*k* = 2)	4.70	1.23	1.67	0.918	0.868	0.577	0.058	0.094	0.853
	RMI (*k* = 3)	4.79	1.25	1.68	0.916	0.863	0.577	0.060	0.095	0.850
	RMI (*k* = 4)	4.90	1.28	1.68	0.914	0.857	0.577	0.062	0.096	0.849
	RMI	4.64	**1.214**	**1.677**	**0.9193**	**0.8734**	0.712	**0.0556**	**0.0919**	**0.8576**
	GSA	6.38	1.66	1.93	0.880	0.846	2.239	0.097	0.143	0.774
	GLP	6.94	1.74	2.43	0.830	0.795	**0.563**	0.167	0.169	0.692
	GLP-H	5.29	1.38	1.72	0.912	0.868	0.574	0.068	0.091	0.847
	EXP	9.63	2.52	2.42	0.661	0.453	0.567	0.000	0.099	0.901
QB	RMI (*k* = 0)	**5.65**	**1.398**	**1.877**	**0.892**	**0.837**	0.717	0.086	0.115	0.809
	RMI (*k* = 1)	5.81	1.43	1.90	0.889	0.833	0.717	0.087	0.116	0.807
	RMI (*k* = 2)	6.02	1.48	1.93	0.886	0.828	0.717	0.088	0.117	0.805
	RMI (*k* = 3)	6.26	1.53	1.97	0.881	0.822	0.717	0.090	0.117	0.803
	RMI (*k* = 4)	6.54	1.60	2.00	0.877	0.814	**0.716**	0.091	0.118	0.802
	RMI	5.66	1.401	1.879	0.890	0.836	0.945	0.087	0.114	0.809
	GSA	7.22	1.73	2.53	0.877	0.797	2.871	**0.045**	**0.086**	**0.872**
	GLP	8.42	2.08	2.88	0.701	0.715	0.737	0.172	0.209	0.655
	GLP-H	6.34	1.54	1.99	0.888	0.832	0.725	0.079	0.102	0.827
	EXP	9.97	2.37	2.97	0.743	0.487	0.717	0.000	0.088	0.912

**Table 2:** Quality indexes for fused products of the improved RMI using different values for *k*.

Image	Method	Degraded Scale					
		RASE	ERGAS	SAM	Q2^n^	SCC	SAM_d_
QB	RMI (*k* = 0)	5.651	1.398	1.877	0.891	0.837	0.7138
	RMI (*k* = 1)	5.812	1.434	1.900	0.889	0.833	0.7138
	RMI (*k* = 2)	6.017	1.480	1.930	0.885	0.828	0.7138
	RMI (*k* = 3)	6.261	1.534	1.965	0.881	0.822	0.7138
	RMI (*k* = 4)	6.541	1.597	2.005	0.877	0.814	0.7138
	RMI (*k* = 5)	6.851	1.667	2.047	0.871	0.805	0.7137
	RMI (*k* = 6)	7.187	1.743	2.093	0.865	0.795	0.7137
	RMI (*k* = 7)	7.547	1.824	2.140	0.859	0.785	0.7137
	RMI (*k* = 8)	7.926	1.910	2.190	0.852	0.774	0.7137
	RMI (*k* = 9)	8.321	2.001	2.241	0.845	0.764	0.7136
	RMI (*k* = 10)	8.731	2.094	2.294	0.837	0.753	0.7136

**Table 3:** Quality indexes for fused products of the proposed method using different values for *S*.

Image	*S*	Degraded Scale				
		RASE	ERGAS	SAM	Q2^n^	SCC
QB	0.1	5.652	1.399	1.877	0.891	0.837
	0.2	5.651	1.398	1.877	0.892	0.837
	0.3	5.651	1.398	1.877	0.891	0.837
	0.4	5.652	1.399	1.878	0.891	0.837
	0.5	5.654	1.399	1.879	0.891	0.836
	0.6	5.658	1.400	1.883	0.890	0.836
	0.7	5.665	1.402	1.888	0.889	0.836
	0.8	5.676	1.404	1.895	0.887	0.836
	0.9	5.693	1.409	1.905	0.885	0.835
	1	5.718	1.415	1.917	0.881	0.834
